# Dual-biomarker strategy for prenatal prediction of ABO hemolytic disease: Maternal sVE-cadherin and anti-A/B IgG titer

**DOI:** 10.1097/MD.0000000000047146

**Published:** 2026-01-09

**Authors:** Dan-Dan Jiang, Jian-Wei Li, Xiang-Chun Zhang

**Affiliations:** a Qingdao West Coast New Area District People’s Hospital, Qingdao, Shandong Province, China; b Qingdao Blood Center, Qingdao, Shandong Province, China.

**Keywords:** anti-A/B IgG, hemolytic disease of the fetus and newborn, maternal, soluble vascular endothelial cadherin

## Abstract

ABO-haemolytic disease of the fetus and newborn remains the leading cause of neonatal hyperbilirubinaemia; however, a reliable antenatal predictor is currently lacking. This study aimed to investigate whether the combination of maternal soluble vascular-endothelial cadherin (sVE-Cadherin) levels with immunoglobulin γ (IgG) anti-A/B titers enhances prenatal detection of ABO-haemolytic disease of the fetus and newborn. We conducted a case-control study involving blood group O Rh(D)-positive mothers, categorized based on the occurrence of ABO hemolytic disease in their newborns. The maternal sVE-cadherin levels and anti-A/B IgG titers in the 2 groups were compared and analyzed. Additionally, we analyzed their correlation with total bilirubin levels in infants and assessed their diagnostic value for the prenatal detection of ABO hemolytic disease. The sVE-cadherin levels and anti-A/B IgG titers of pregnant women in the ABO hemolytic disease group were significantly higher than those observed in the control group (*P* < .001). A positive correlation was found between sVE-cadherin levels and total bilirubin (*R*^2^ = 0.503, *P* < .001) in the ABO hemolytic disease cohort. The area under the curve (AUC) for the sVE-cadherin level was 0.844, with a sensitivity of 84.91% and specificity of 70.00%. For the anti-A/B IgG titer, the AUC was 0.897, with a sensitivity of 96.23% and specificity of 72.73%. When combined for diagnosis, these metrics yielded an AUC of 0.923, a sensitivity of 95.24%, and a specificity of 86.84%. Maternal sVE-cadherin levels along with anti-A/B IgG titers provide significant reference values for prenatal diagnosis of ABO hemolytic disease; together, they demonstrate excellent joint diagnostic efficacy.

## 1. Introduction

Hemolytic disease of the fetus and newborn (HDFN) generally refers to a spectrum of symptoms arising from incompatibility between maternal and infant blood groups, specifically due to the presence of immunoglobulin γ (IgG) antibodies against fetal red blood cell antigens in the mother.^[1,2]^ With a significant decline in cases of maternal-fetal Rh incompatibility, ABO incompatibility has emerged as the most prevalent cause of HDFN, with severe cases reported intermittently.^[3,4]^

Maternal alloantibodies belonging to the IgG class can traverse the placenta and lead to the destruction of fetal cells through phagocytosis in both fetuses and newborns, resulting in HDFN.^[5]^ In our study, we observed that among newborns born to O-type Rh(D) positive mothers, 17.65% developed ABO-Hemolytic disease of the fetus and newborn (ABO-HDFN). Among those diagnosed with ABO-HDFN, 66.04% required phototherapy, 30.19% required both phototherapy and gamma-globulin therapy, and 3.77% required exchange transfusion. Research underscores the importance of regular monitoring of maternal IgG anti-A/B antibody levels prior to delivery.^[6]^ However, the correlation between neonatal hemolysis severity and serum antibody titers in pregnant women remains an ongoing debate.^[7]^

ABO antigens are expressed not only on the surfaces of red blood cells but also in various other tissues, particularly within the vascular endothelium.^[8,9]^ Previous studies have demonstrated that vascular endothelial damage occurs in neonates with ABO-HDFN, as evidenced by measurements of endothelial microparticles (EMPs).^[10]^ This injury is associated with both the occurrence and severity of hemolytic diseases.^[11]^ Vascular endothelial cadherin (VE-cadherin) is a membrane protein that serves as a primary component of adherens junctions between endothelial cells,^[12]^ playing a crucial role in maintaining vascular integrity, regulating endothelial permeability, and facilitating angiogenesis.^[13]^ Soluble VE-cadherin acts as a marker for disruption of these junctions, which can lead to capillary leakage, tissue edema, and organ dysfunction.

Consequently, the author sought to investigate whether the concentrations of markers indicative of vascular endothelial injury, such as soluble VE-cadherin, in maternal peripheral blood would be influenced by placental transfer during intrauterine onset.

In this study, correlation analyses were performed to assess the relationship between maternal peripheral blood levels of soluble VE-cadherin and IgG anti-A/B titers during late pregnancy, concerning the occurrence and progression of ABO-HDFN. This study aimed to provide a theoretical basis for prenatal diagnosis, along with early prevention and treatment strategies for ABO-HDFN.

## 2. Subjects and methods

### 2.1. General clinical data of study patients

We conducted a case-control study involving 103 pregnant women with a type O Rh(D) positive blood group, who were examined at the Huangdao District People’s Hospital between April 2020 and February 2023. The study population was divided into 2 groups, the control group (n = 50) and the ABO-HDFN group (n = 53), whose newborns were diagnosed with ABO-HDFN.

### 2.2. Inclusion and exclusion criteria

- Inclusion criteria:
  - Pregnant women aged 20 to 46 years.
  - Gestational age ≥ 38 weeks.
  - Negative irregular antibody screening during pregnancy.
  - Husbands with RhD-positive non-type O blood groups (55 cases in blood group A, 47 cases in blood group B, and 1 case in blood group AB).
  - Normal liver and kidney function.
- Exclusion criteria:
  - History of blood transfusions.
  - Chronic hypertension, diabetes, systemic lupus erythematosus, and other autoimmune diseases.

### 2.3. Diagnostic criteria for ABO-HDFN

1. Maternal-fetal ABO incompatibility.
2. Hyperbilirubinemia.
3. Simultaneous positivity in both the direct antiglobulin test and serum antibody test or a positive result in the elution test.
4. Other conditions, such as sepsis, neonatal hypoxia, large cephalohematoma, severe contusions, congenital malformations, or hemolysis due to alternative blood group systems and other causes, include thalassemia, hereditary spherocytosis, and glucose-6-phosphate dehydrogenase deficiency.

### 2.4. Diagnostic criteria for neonatal hyperbilirubinemia

1. Total bilirubin (TBIL) level > 103 µmol/L at 24 hours
2. TBIL > 154 µmol/L at 48 hours
3. TBIL > 220.6 µmol/L after 3 days
4. Predominantly elevated indirect bilirubin level

## 3. Measurements

### 3.1. Blood sample collection

Peripheral venous blood or umbilical cord blood was collected into ethylenediaminetetraacetic acid (1.2 mg/mL) for hematological profiling, irregular antibody screening, 3 hemolytic disease tests (direct antiglobulin test, free antibody test, and elution test), measurement of IgG antibody titers, and soluble vascular endothelial cadherin (sVE-cadherin) levels. Clotted samples were obtained for TBIL, lactate dehydrogenase (LDH), and C-reactive protein assays.

### 3.2. Irregular antibody screening and blood group identification

The IH1000 automatic blood group analyzer was used with microcolumn gel cards. All steps, including dilution, sample addition, incubation, centrifugation, and result interpretation, were automatically completed by the instrument, following standard operating procedures. C-reactive protein and neonatal chemical analyses were performed using Cobas Integra 6000 (Roche Diagnostics, Mannheim, Germany).

### 3.3. IgG anti-A/B titer measurement

In the third trimester, 100 µL of maternal plasma was mixed with an equal volume of β-mercaptoethanol and incubated at 37°C for 10 minutes to destroy the IgM antibodies. Serial dilutions of maternal plasma samples were prepared in phosphate-buffered saline at the following dilutions: 1:8, 1:16, 1:32, 1:64, 1:128, 1:256, and 1:512. A1 and B red blood cells (Shanghai Blood Biological Medicine Co., China), and maternal plasma were added to the LISS/Coombs card containing anti-IgG (DiaMed, ID-cards no.50531, Cressier, Switzerland). After incubation at 37°C for 30 minutes, the IgG antibody titer of the corresponding blood group was determined.

### 3.4. sVE-cadherin measurement

sVE-cadherin levels in the maternal blood were determined using the double antibody one-step sandwich method (human vascular endothelial cadherin (VE-CAD) Enzyme-linked immunosorbent assay kit, JL19938, Jianglaibio, China) in the third trimester. Samples, standard samples, and HRP-labeled antibodies were added to the pre-coated micropores of the antibodies captured by endothelial cadherin, incubated at 37°C for 60 minutes, and washed thoroughly. The TMB substrate was colored, turning blue under peroxidase catalysis and yellow under acid action. The absorbance (OD value) was measured at 450 nm using an enzyme-labeled analyzer (Hengmei, HM-SY96S, China), and the concentration of the sample was calculated.

### 3.5. Statistical analysis

The measurement data were assessed for normality using the Kolmogorov–Smirnov test, and normally distributed indicators were expressed as mean ± standard deviation (mean ± SD). Intergroup comparisons were conducted using an independent-sample *t*-test. Indicators that did not follow a normal distribution were represented by median and interquartile range (IQR), with the Mann–Whitney *U* test employed for inter-group comparisons, while the Kruskal–Wallis *H* test was used for multiple group comparisons. Statistical data are presented as percentages (%), and group comparisons were performed using either the χ^2^ test or Fisher exact probability method. Spearman correlation analysis and multivariate correction were used to evaluate the relationship between the 2 factors. Diagnostic efficiency was assessed using a receiver operating characteristic curve analysis. To determine the cutoff values for IgG titer and sVE-cadherin as predictors of blood component transfusion in infants with ABO hemolytic disease of the newborn (ABO-HDN), we calculated the Youden index. The Youden index was reported along with the corresponding sensitivity and specificity values for IgG titer and sVE-cadherin levels. A *P*-value of <.05 was considered indicative of a statistically significant difference in all analyses.

## 4. Results

**Figure 1.**
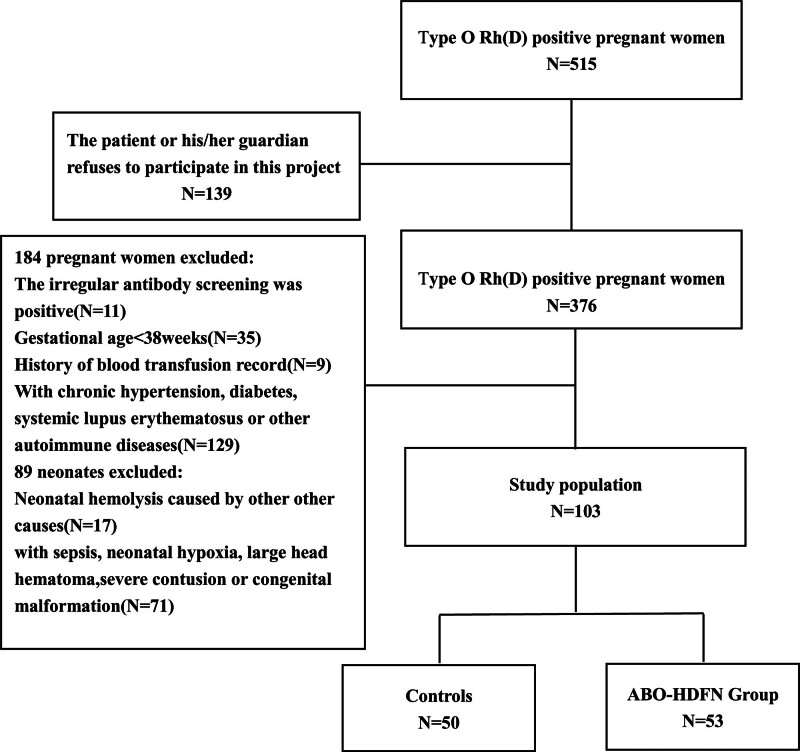
Flowchart of the study population.

During the study period, 515 pregnant women with type O Rh(D)-positive blood underwent examination at our hospital before giving birth. A total of 412 cases were excluded: 139 individuals declined participation in the study, 184 were excluded during pregnancy, and 89 were excluded postnatally. The derivation of the study population is shown in Figure 1.

The clinical data pertaining to both pregnant women and their newborns are summarized in Table 1. No statistically significant differences emerged between the ABO-HDFN group and the control group regarding maternal age, gravidity, birth weight, or sex of newborns (*P* > .05). However, hemoglobin (Hb) levels, reticulocyte percentage (RET%), and markers of hemolysis –TBIL and LDH – were found to be significantly different between neonates in the ABO-HDFN groups compared to controls (*P* < .05).

**Table 1:** Baseline characteristics of study population.

Variables	ABO-HDFN	Controls	χ^2^/ *U*/*t*	*P*-value
	n = 53 (51.46%)	n = 50 (48.54%)		
Maternal age (yr) n (%)				
20–29	21 (39.62)	27 (54.00)	2.865	.239
30–39	30 (56.60)	20 (40.00)		
≥40	2 (3.77)	3 (6.00)		
Gravidity n (%)				
≤1	16 (30.19)	17 (34.00)	0.041	.839
>1	37 (69.81)	33 (66.00)		
Delivery n (%)				
Cesarean	24 (45.28)	26 (52.00)	0.235	.628
Normal	29 (54.72)	24 (48.00)		
Sex of newborn n (%)				
Male	24 (42.86)	23 (44.74)	0.016	.901
Female	29 (57.14)	27 (55.26)		
Neonatal blood type n (%)				
A	26 (49.06)	30 (60.00)	0.840	.359
B	27 (50.94)	20 (40.00)		
Birth weight (g) (mean ± SD)	3541 ± 450.09	3513 ± 418.11	0.327	.744
Test day of life for newborns (d) (median (IQR))	1.00 (0.50–2.00)	0.50 (0.50–2.50)	1185.000	.940
Hb (g/L) of neonate (median (IQR))	172.50 (158.00–179.00)	181.00 (171.00–189.00)	1688.000	<.001
RET (%) of neonate (median (IQR))	3.95 (2.77–5.76)	2.99 (1.98–4.56)	800.500	.007
TBIL (µmol/L) of neonate (median (IQR))	190.90 (159.05–221.55)	114.80 (90.10–125.40)	132.000	<.001
LDH (U/L) of neonate (median (IQR))	562.50 (361.50–786.75)	304.00 (193.00–406.00)	526.000	<.001

Values are expressed as number (%), mean ± standard deviation (SD), and median with interquartile range (IQR).

ABO-HDFN = ABO-hemolytic disease of the fetus and newborn, Hb = hemoglobin, LDH = lactate dehydrogenase, RET = reticulocyte, TBIL = total bilirubin.

**Figure 2.**
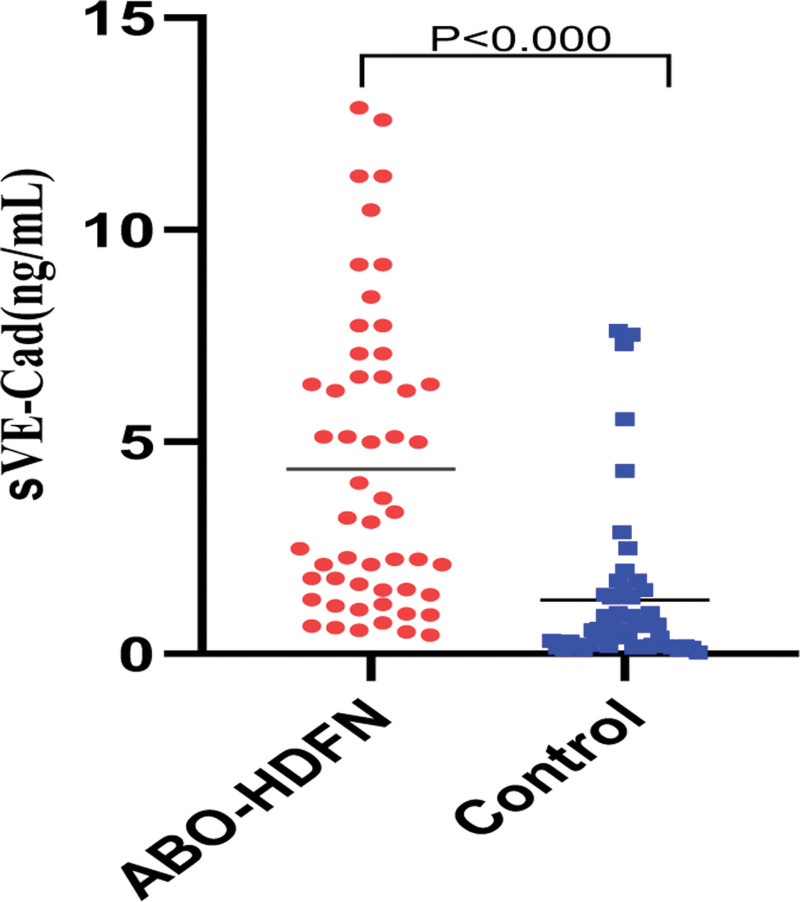
Comparison of sVE-Cad levels in pregnant women in the ABO-HDFN and control groups. ABO-HDFN = ABO-hemolytic disease of the fetus and newborn, sVE-Cad = soluble vascular endothelial cadherin.

Compared to the control group, the levels of sVE-Cad in pregnant women were significantly elevated in the ABO-HDFN group (4.36 ng/mL [0.65–12.88] vs 1.27 ng/mL [0.11–7.54], *P* < .001), as illustrated in Figure 2.

In late pregnancy, there were 48 cases (90.57%) with IgG anti-A/B titers ≥ 1:64 in the ABO-HDFN group; conversely, only 18 cases (36.00%) reached this threshold in the control group (Table 2).

**Table 2:** Distribution of antibody titers in pregnant women (n(%)).

	≤1:32	1:64	1:128	1:256	≥1:512	χ^2^	*P*-value
ABO-HDFN	2 (3.77)	4 (7.55)	7 (13.21)	17 (32.08)	23 (43.39)	48.80	<.0001
Control	32 (64.00)	5 (10.00)	5 (10.00)	5 (10.00)	3 (6.00)		

ABO-HDFN = ABO-hemolytic disease of the fetus and newborn.

**Figure 3.**
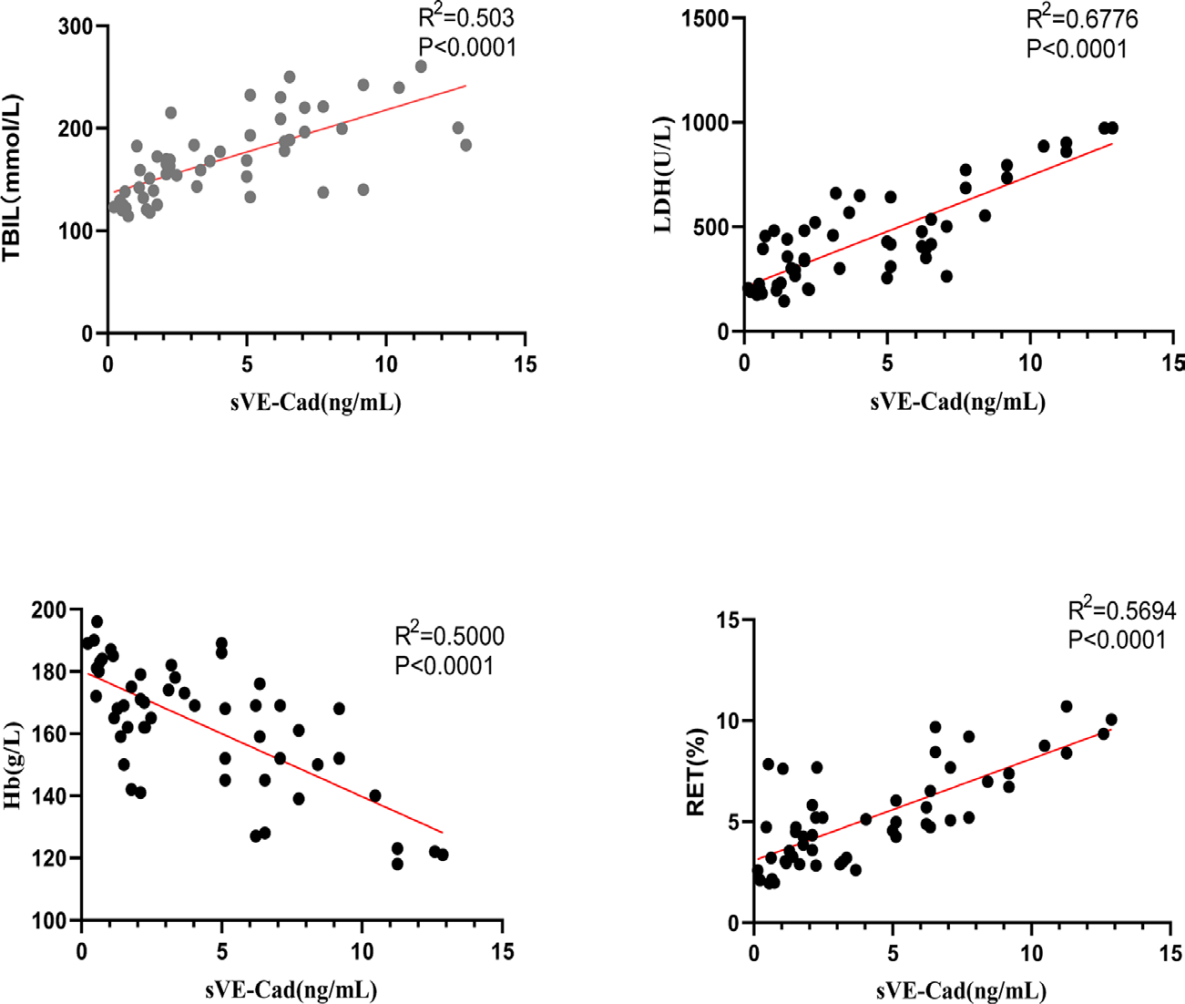
Correlation analysis of sVE-Cad levels with neonatal TBIL, LDH, Hb, and RET%. Hb = hemoglobin, LDH = lactate dehydrogenase, RET% = reticulocyte percentage, sVE-Cad = soluble vascular endothelial cadherin, TBIL = total bilirubin.

Furthermore, significant positive correlations were found between sVE-Cad levels and neonatal TBIL (*R*^2^ = 0.503, *P* < .0001), LDH (*R*^2^ = 0.6776, *P* < .0001), Hb (*R*^2^ = 0.5000, *P* < .0001), and RET% (*R*^2^ = 0.5694, *P* < .0001) (Fig. 3). Multiple regression analysis showed that TBIL, LDH, Hb, and RET% in the ABO hemolytic group were independently correlated with sVE-Cad levels (all *P* < .05) (Table 3).

**Table 3:** Multiple regression analysis of the relationship between sVE-Cad levels and laboratory variables in the ABO-HDFN group.

Variables	Unstandardized coefficients		Standardized coefficients	*T*	*P*	95.0% CI for *B*	
	*B*	SE	Beta			Lower limit	Upper limit
TBIL	0.051	0.016	0.362	3.173	.003	0.019–0.084	
LDH	0.006	0.002	0.232	2.558	.014	0.001–0.010	
Hb	−0.097	0.036	−0.274	−2.681	.010	−0.169–−0.024	
RET%	0.881	0.338	0.333	2.606	.012	0.200–1.562	

ABO-HDFN = ABO-hemolytic disease of the fetus and newborn, CI = confidence interval, Hb = hemoglobin, LDH = lactate dehydrogenase, RET% = reticulocyte percentage, sVE-Cad = soluble vascular endothelial cadherin, TBIL = total bilirubin.

**Figure 4.**
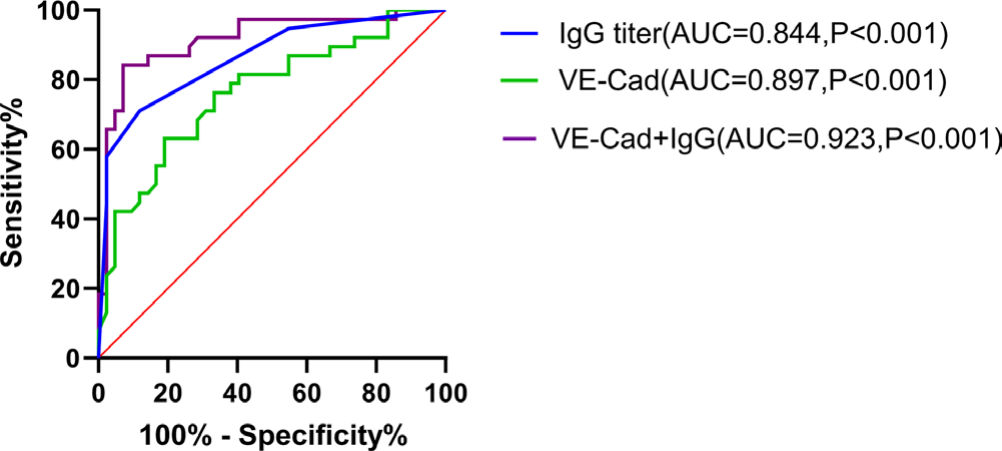
ROC curve analysis of sVE-Cad level and IgG antibody titer alone and their combined diagnostic value. IgG = immunoglobulin γ, ROC = receiver operating characteristic curve, sVE-Cad = soluble vascular endothelial cadherin.

Receiver operating characteristic curve analysis of the diagnostic value of sVE-Cad level and IgG anti-A/B titers showed that the area under the curve (AUC) of sVE-Cad level was 0.762, with a cutoff value (cutoff) of 1.008 ng/mL, sensitivity of 80.95%, and specificity of 63.16%; the AUC of IgG antibody titer was 0.862, with a cutoff value of 1:64, sensitivity of 88.10%, and specificity of 71.05%. When combining both diagnostic measures, an AUC of 0.915 was achieved, with sensitivities and specificities of 92.86% and 84.21%, respectively (Fig. 4, Table 4).

**Table 4:** Area under the curve of sVE-Cad level, IgG anti-A/B titer, and combination factor.

	AUC	Standard deviation*	*P* value†	95% CI	
				Lower limit	Upper limit
sVE-Cad	0.843	0.039	<.0001	0.768	0.919
IgG anti-A/B titer	0.897	0.033	<.0001	0.832	0.963
combination factor	0.923	0.029	<.0001	0.894	0.989

AUC = area under the curve, IgG = immunoglobulin γ, CI = confidence interval sVE-Cad = soluble vascular endothelial cadherin.

* Nonparametric assumption.

† Null hypothesis: true area = 0.5.

## 5. Discussion

HDFN is most common in the ABO blood group. The underlying mechanism involves the entry of incompatible fetal red blood cells into maternal circulation through fetomaternal hemorrhage, which stimulates the mother to produce IgG antibodies. These antibodies subsequently cross the fetal circulation and bind to fetal red blood cells, leading to their destruction.^[14]^

Clinical manifestations of HDFN include fetal edema, early abortion or stillbirth, neonatal jaundice, anemia, hepatosplenomegaly, nuclear jaundice, and even death. Evidence suggests that IgG blood-group antibodies produced by the mother can also bind to the blood-group antigens present on vascular endothelial surfaces, resulting in endothelial damage.^[15]^ However, few studies have investigated the markers of vascular endothelial injury in patients with HDFN;this gap affects clinical treatment choices and assessments of therapeutic efficacy.

Numerous studies have indicated a positive correlation between maternal IgG anti-A/B antibody levels and the incidence of neonatal hemolytic disease. Clinically, a maternal serum antibody titer threshold of 1:64 is typically regarded as critical; titers at or above this level suggest a potential risk of ABO-HDFN in infants.^[16]^ Nonetheless, relying solely on prenatal detection of maternal serum IgG anti-A(B) antibody titers to predict perinatal hemolysis presents certain limitations. In this study, 51 of 53 patients (96.23%) in the ABO-HDFN group exhibited an antibody titer ≥ 1:64, consistent with relevant literature reports.^[17]^ Notably, there were also 2 cases with a titer ≤ 1:32, highlighting that hyperbilirubinemic infants with low maternal antibody titers should not be overlooked. Conversely, in the control group, those with an antibody titer ≥ 1:64 accounted for 36.00%. Among these cases, 3 individuals with an antibody titer ≥ 1:512 did not develop HDFN, and no significant correlation was found between IgG anti-A/B titers and neonatal TBIL levels (*P* > .05). This lack of correlation may be attributed to factors such as antibody subtype variations, differences in blood group substance content, placental effects, and other influencing elements.^[18]^

ABO blood group antigens are tissue-specific antigens that are widely distributed across human tissues and organs and are prominently expressed on the surface of endothelial cells. Consequently, antigen-antibody reactions occur not only on red blood cell surfaces, but also on endothelial cell surfaces, resulting in more severe and direct damage to blood vessels in cases of ABO-HDFN.^[19]^

Vascular endothelial cadherin (VE-Cad) is a critical structural molecule that facilitates the adhesion and connectivity among vascular endothelial cells. It plays a vital role in maintaining the stability of the vascular endothelial barrier by interacting with connexins and actin cytoskeleton proteins, making it an essential component for vascular development and the regulation of permeability.^[20]^ VE-Cad can function via 3 primary mechanisms: complete endocytosis, shedding of the extracellular domain, or redistribution within the cell membrane. Given that direct assessment of intact cadherin endocytosis or intracellular redistribution is not feasible in clinical settings, soluble VE-Cad shed into the patient’s circulation may serve as a biomarker for the disruption of endothelial junctions, which can lead to capillary leakage, tissue edema, and organ dysfunction.^[21]^ Studies have demonstrated that inflammatory mediators and growth factors, including lipopolysaccharides, tumor necrosis factor, and vascular endothelial growth factor, can concurrently induce various alterations in soluble VE-Cad levels. Elevated plasma levels of sVE-Cad have been observed in conditions such as sepsis, malignancy, autoimmune diseases, and coronary atherosclerosis.^[22]^

In our previous study, we found that increased VE-Cad levels in newborns with ABO hemolytic disease could reflect endothelial injury and help assess the risk of severe vascular dysfunction.^[23]^ VE-Cad is a small molecule (approximately 30.5 kDa) that can cross the placenta freely. Therefore, we hypothesized that sVE-Cad levels in the maternal peripheral blood might be affected by fetal intrauterine onset.

In this study, we detected and analyzed sVE-Cad levels in the maternal blood during the third trimester. The results showed that sVE-Cad levels were significantly higher in the ABO-HDFN group (*P* < .0001) and positively correlated with neonatal TBIL, LDH, and RET% levels (*R*^2^ = 0.503, 0.6776, and 0.5694, respectively, all *P* < .0001). This suggests that elevated sVE-Cad levels may be associated with endothelial injury in ABO-HDFN and could effectively predict hemolysis severity.

Additionally, the combined detection of sVE-Cad levels and IgG anti-A/B antibody titers demonstrated superior diagnostic efficacy compared with either marker alone. The AUC, sensitivity, and specificity of the combined diagnosis were 0.915, 92.86%, and 84.21%, respectively. In contrast, the AUC, sensitivity, and specificity of sVE-Cad levels alone were 0.762, 80.95%, and 63.16%, respectively, whereas those of IgG anti-A/B antibody titers were 0.862, 88.10%, and 71.05%, respectively. This indicates that combining sVE-Cad levels and IgG anti-A/B antibody titers can significantly improve the accuracy of prenatal diagnosis of ABO-HDFN.

This study has several limitations. First, the sample size was modest. Second, we did not quantify additional soluble endothelial markers – such as vascular cell adhesion molecule-1, intercellular adhesion molecule-1, and P-selectin – that could have provided further insight into endothelial injury. Third, we did not prospectively track post-treatment VE-cadherin levels in the enrolled children. Future work should therefore expand the cohort and incorporate a broader panel of endothelial-injury biomarkers to yield more comprehensive and clinically relevant data.

On the basis of these findings, we recommend that all women planning a pregnancy undergo ABO blood-group typing and quantitative IgG anti-A/B antibody screening either pre-conception or at the first prenatal visit (8–12 weeks). If the IgG anti-A/B titer is <1:64, repeat testing should be scheduled after 28 weeks. When the titer remains unchanged, routine obstetric care and vaginal delivery can be anticipated. If the titer is ≥1:64 or rises by ≥2 serial dilutions (e.g., from 1:4 to 1:16), surveillance should be intensified to every 2 weeks from 28 weeks until delivery. In parallel, serial measurement of circulating VE-cadherin levels can be added to improve the antenatal detection rate of ABO-HDFN.

## 6. Conclusion

The levels of sVE-Cad and IgG antibodies against A/B antigens in pregnant women in the third trimester are elevated in patients with ABO-HDFN. Combining the detection of sVE-Cad levels and IgG anti-A/B antibody titers provides a more effective approach for prenatal diagnosis of ABO-HDFN than using either marker alone. This combined approach can enhance the accuracy of prenatal diagnoses and facilitate better clinical management. Future studies should explore additional soluble endothelial markers to further improve the diagnostic accuracy.

## Acknowledgments

The authors are grateful to the editors and peer reviewers for their constructive suggestions, which significantly improved the quality of this paper.

## Author contributions

**Data curation:** Dan-Dan Jiang, Jian-Wei Li, Xiang-Chun Zhang.

**Formal analysis:** Xiang-Chun Zhang.

**Investigation:** Dan-Dan Jiang, Jian-Wei Li.

**Writing – original draft:** Dan-Dan Jiang, Xiang-Chun Zhang.
